# LEVERAGING PROJECT ECHO FOR EVIDENCE-BASED INFECTION CONTROL IN NURSING HOMES: A RANDOMIZED CONTROLLED TRIAL

**DOI:** 10.1093/geroni/igad104.2023

**Published:** 2023-12-21

**Authors:** Jennifer Kraschnewski, Lan Kong, Erik Lehman, Emily Heilbrunn, Liza Behrens, William Calo

**Affiliations:** Penn State College of Medicine, Hershey, Pennsylvania, United States; Penn State College of Medicine, Hershey, Pennsylvania, United States; Penn State College of Medicine, Hershey, Pennsylvania, United States; Penn State College of Medicine, Hershey, Pennsylvania, United States; Penn State Ross and Carol Nese College of Nursing, State College, Pennsylvania, United States; Penn State College of Medicine, Hershey, Pennsylvania, United States

## Abstract

Nursing homes were ill-equipped for the pandemic; though facilities are required to have infection control staff, only 3% have taken a basic infection control course. Significant research has focused on infection control in the acute care setting; however, little is known about the implementation of practices and effective interventions. We proposed an intervention utilizing Project ECHO to connect Penn State University experts with remote nursing home staff and administrators to support evidence-based infection control guideline implementation. This study sought to answer the critical research question of how evidence-based infection control guidelines can be implemented effectively in nursing homes. A stratified cluster randomized design was used to randomize nursing homes from the Northeast and Midwest to either: 1) AHRQ-funded COVID-19 ECHO that included 16 weekly tele-mentoring sessions addressing COVID-19 guidelines and best practices plus 9 optional, weekly 60-minute office hour sessions, or 2) AHRQ-funded COVID-19 ECHO+, which included 16 weekly tele-mentoring sessions addressing COVID-19 guidelines and best practices, plus 17 weekly 60-minute sessions with a focus on stakeholder-identified needs and CDC infection control training. A total of 136 nursing homes were recruited. There were no statistically significant differences in COVID-19 infection rate, hospitalization, deaths, or flu-like illness. A multipronged approach to improving infection control and emergency preparedness in nursing homes is important. The ECHO model has significant strengths when compared to traditional training, as it allows for remote learning delivered by a multidisciplinary team of experts and utilizes case discussions that match the context and capacity of nursing homes.

